# Assessment of CD4+ T Cell Responses to Glutamic Acid Decarboxylase 65 Using DQ8 Tetramers Reveals a Pathogenic Role of GAD65 121–140 and GAD65 250–266 in T1D Development

**DOI:** 10.1371/journal.pone.0112882

**Published:** 2014-11-18

**Authors:** I-Ting Chow, Junbao Yang, Theresa J. Gates, Eddie A. James, Duy T. Mai, Carla Greenbaum, William W. Kwok

**Affiliations:** 1 Benaroya Research Institute at Virginia Mason, Seattle, WA, United States of America; 2 Department of Medicine, University of Washington, Seattle, WA, United States of America; La Jolla Institute for Allergy and Immunology, United States of America

## Abstract

Susceptibility to type 1 diabetes (T1D) is strongly associated with MHC class II molecules, particularly HLA-DQ8 (DQ8: DQA1*03:01/DQB1*03:02). Monitoring T1D-specific T cell responses to DQ8-restricted epitopes may be key to understanding the immunopathology of the disease. In this study, we examined DQ8-restricted T cell responses to glutamic acid decarboxylase 65 (GAD65) using DQ8 tetramers. We demonstrated that GAD65_121–140_ and GAD65_250–266_ elicited responses from DQ8+ subjects. Circulating CD4+ T cells specific for these epitopes were detected significantly more often in T1D patients than in healthy individuals after in vitro expansion. T cell clones specific for GAD65_121–140_ and GAD65_250–266_ carried a Th1-dominant phenotype, with some of the GAD65_121–140_-specific T cell clones producing IL-17. GAD65_250–266_-specific CD4+ T cells could also be detected by direct ex vivo staining. Analysis of unmanipulated peripheral blood mononuclear cells (PBMCs) revealed that GAD65_250–266_-specific T cells could be found in both healthy and diabetic individuals but the frequencies of specific T cells were higher in subjects with type 1 diabetes. Taken together, our results suggest a proinflammatory role for T cells specific for DQ8-restricted GAD65_121–140_ and GAD65_250–266_ epitopes and implicate their possible contribution to the progression of T1D.

## Introduction

Type 1 diabetes (T1D) results from destruction of the insulin-producing beta cells of the pancreas. A number of genes have been implicated in T1D development, but genes within the HLA class II region confer most of the disease risk [1]; in particular, it is estimated that 90% of T1D subjects have either an HLA-DQ8 or DQ2 allele [2]. Subjects with the DRB1*04:01 (DR0401)-DQ8 haplotype have an odds ratio of 8.4 for T1D [3], and the predisposing effect of this haplotype is supported by meta-analysis of data sets from different geographic regions [4]. In accordance with the importance of MHC class II molecules in antigen presentation, DQ8-restricted CD4+ T cells are likely to have an essential and pathogenic role in the progression of T1D.

A special feature of DQ8 is the absence of an aspartic acid residue at position 57 of the beta chain [5]. Lack of this Asp residue at beta 57 leads to reduced affinity for antigenic peptides, giving rise to diabetic pathology as a result of ineffective tolerance induction in the thymus [6], [7]. Identification and characterization of DQ8-restricted self-epitopes may be key to comprehending DQ8-mediated autoimmunity. A number of DQ8-restriced self-epitopes have been identified using DQ8 transgenic mice (for a recent review see [8]). Some of the reported peptides also elicit responses in HLA-DQ8+ individuals. However, monitoring of specific CD4+ T cells during the progression of diabetes, especially direct assessment of responses in the periphery, has been hampered by low frequencies of effector cells [9] and heterogeneity of the disease.

Autoantibodies against insulin (IA), glutamic acid decarboxylase 65 (GAD65), islet tyrosine phosphatase (IA-2), and zinc transporter 8 (ZnT8) have been used as predictive markers for T1D [10]–[13]. The appearance of autoantibodies is clear indication of beta cell autoimmunity and the combined measurement of insulin, GAD65, IA-2, and ZnT8 autoantibodies raise the detection rate to 98% at disease onset [12]. Despite their predictive value, some people with autoantibodies never develop diabetes [14]. In addition, the presence of autoantibodies does not necessarily indicate insulitis, the histopathologic hallmark of T1D that is mainly mediated by T-lymphocytes [15], [16]. Therefore, identification of T cell biomarkers correlated with pathogenesis of T1D, together with the presence of autoantibodies, will facilitate disease prediction and prevention.

In this study, we investigated DQ8-restricted CD4+ T cell responses to GAD65. Responses of CD4+ T lymphocytes from T1D subjects to GAD65_121–140_ and GAD65_250–266_ were visualized using DQ8 tetramers after in vitro expansion of antigen-specific cells with the relevant peptides. In vitro responses and intracellular cytokine staining suggested a strong association of GAD65_121–140_ and GAD65_250–266_ to the disease. Direct ex vivo staining of peripheral blood mononuclear cells (PBMCs) for GAD65_250–266_ revealed higher frequencies of CD4+ and CD4+CD45RO+ T cells in the T1D group. Collectively, our work indicates that T cells specific for GAD65_121–140_ and GAD65_250–266_ may contribute to the pathogenesis of T1D, and suggests GAD65_250–266_–specific T cells as a potential biomarker for T1D.

## Results

### CD4+ T cell responses to GAD65_121–140_ and GAD65_250–266_ are detected more often in subjects with type 1 diabetes after in vitro expansion

Seven peptides derived from GAD65 were selected in this study to evaluate their relevance to the progression of autoimmune diabetes. All seven peptides are immunogenic in DQ8 transgenic mice [17]–[19], but DQ8-restricted responses in human subjects were not well demonstrated. Among the seven peptides, GAD65_121–140_, GAD65_206–220,_ GAD65_250–266_ bound to DQ8 with strong affinities (Table 1). The IC_50_ values of GAD65_121–140_, GAD65_206–220,_ and GAD65_250–266_ were comparable to that of influenza A/Puerto Rico/8/34 Matrix Protein M1 (H1MP_185–204_) epitope.

**Table 1:** List of DQ8-restricted GAD65 peptides used in this study.

Peptide	IC_50_ (µM)	Sequence
GAD65 121–140	1.6	YVVKS**FDRSTKVID**FHYPNE
GAD65 176–190	>50	PRY**FNQLSTGLD**MVG
GAD65 206–220	1.5	TYE**IAPVFVLLE**YVT
GAD65 250–266	2.3	AMM**IARFKMFPE**VKEKG
GAD65 427–441	>50	FQQDKH**YDLSYDTGD**
GAD65 461–475	>50	AKG**TTGFEAHVD**KCL
GAD65 508–521	>50	YIPPS**LRTLEDNEE**

*IC_50_ value for the control H1MP_185–204_ peptide was 1.4 µM.

*Predicted binding registers are indicated in boldface.

**Figure 1:**
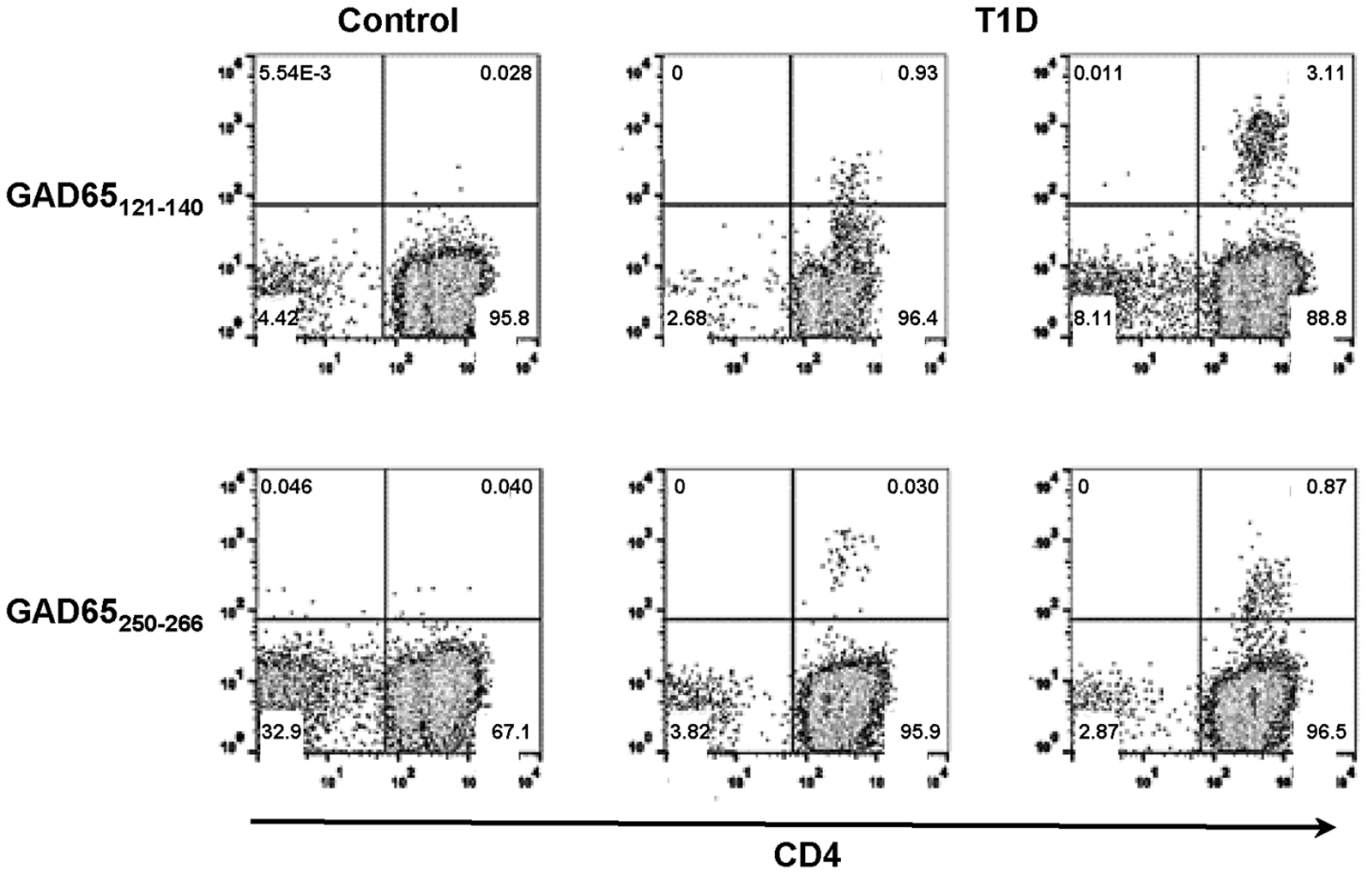
Detection of DQ8-specific self-reactive T cells in T1D patients. CD4+ T cells from T1D patients were stimulated with self-peptides derived from GAD65. The cells were cultured for two weeks and stained with PE-tetramers. CD4+ T cell responses to GAD65_121–140_ and GAD65_250–266_ were detected in multiple subjects. Representative tetramer staining of DQ8-specific self-reactive T cells was shown. Left column: negative staining from a healthy control. Middle and right columns: positive staining from two different T1D patients. Subjects were considered tetramer-positive if a distinct population that was more than three-fold above background in the same experiment was labeled.

The three strong DQ8 binders were used to stimulate CD4+ T cells from DQ8+ T1D subjects. After two-week stimulation, GAD65_121–140_ and GAD65_250–266_ elicited CD4+ T cell responses in multiple subjects. Examples of positive tetramer staining for the two epitopes that elicited responses are shown in Fig. 1. Responses to GAD65_206–220_ were negative in five T1D subjects examined and were not further investigated. The association of GAD65_121–140_ and GAD65_250–266_ with T1D was next examined by comparing the prevalence of specific CD4+ T cell responses in T1D patients and healthy controls. GAD65_121–140_- and GAD65_250–266_-specific CD4+ T cell responses were detected more often in T1D subjects than controls (GAD65_121–140_: 6/10 T1D and 0/9 controls (*p* = 0.0108); GAD65_250–266_: 9/17 T1D and 1/11 controls (Table 2, *p* = 0.0407); *p* value evaluated by two-tailed Fisher's exact tests), demonstrating a clear association of DQ8-restricted GAD65 T cell responses with disease.

**Table 2:** Prevalence of GAD65-specific T cells in T1D and healthy subjects.

	Responses			
	+	-	Total	*P*
GAD65 121–140				
T1D	6	4	10	0.0108
Control	0	9	9	
Total	6	13	19	
GAD65 250–266				
T1D	9	8	17	0.0407
Control	1	10	11	
Total	10	18	28	

Not all peptides were tested on each subject.

Statistical analysis was performed using two-tailed Fisher's exact tests.

### DQ8-restricted self-epitopes elicit pathogenic responses

**Figure 2:**
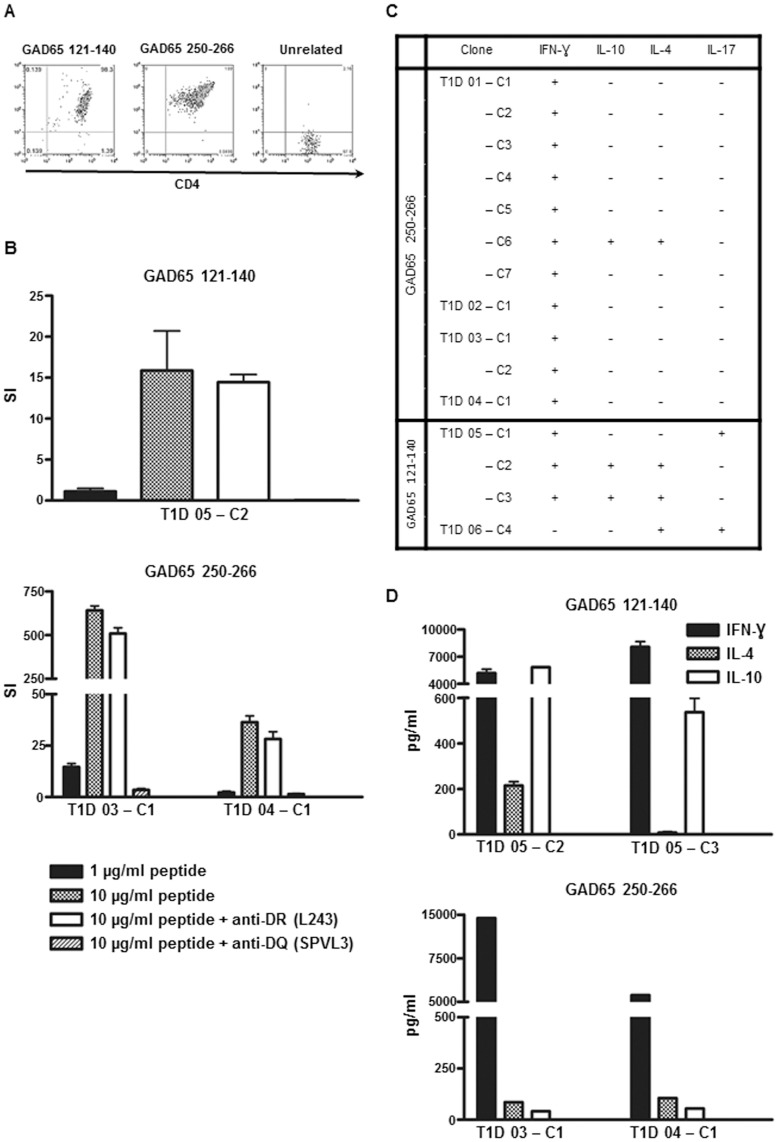
Functional analysis of DQ8-specific self-reactive T cells. T cell clones isolated by DQ8 tetramers were assayed for specificity and functionality. (**A**) Tetramer staining for GAD65_121–140_-specific (clone T1D05-C2), GAD65_250–266_-specific (clone T1D04-C1) and unrelated T cell clones. (**B**) Representative proliferation results of one GAD65_121–140_- and two GAD65_250–266_-specific T cell clones using APCs from a DR0401/DQ8 homozygous individual. Cells were stimulated with specific or irrelevant control peptide in the absence or presence of 20 µg/ml of L243 (HLA-DR blocking antibody) or SPVL3 (HLA-DQ blocking antibody). SI: stimulation index; cpm of specific peptide divided by cpm of irrelevant peptide. (**C**) Summary of cytokine-positive clones (for definition see Materials and methods) for GAD65_121–140_ and GAD65_250–266_. T cell clones were stimulated with 50 ng/mL phorbol 12-myristate 13-acetate and 1 µg/mL ionomycin in the presence of 10 µg/mL Brefeldin A in 1 mL of T cell medium for 4 hours at 37°C. Cells were fixed, permeabilized, stained with antibodies for IFN-γ, IL-10, IL-4, and IL-17, and analyzed on a LSRII multicolor flow cytometer. (**D**) Secretion of IFN-γ, IL-4, and IL-10 from two GAD65_121-140_-specific and two GAD65_250–266_-specific T cell clones. Clones were stimulated in the presence of 10 µg/ml of specific peptide with DQ8 antigen presenting cells.

Self-peptides can modulate autoimmunity by induction of pathogenic (Th1/Th17) or suppressive (Th2/Treg) responses. To examine functional properties of DQ8-restricted self epitopes, GAD65_121–140_- and GAD65_250–266_-specific CD4+ T cells were cloned from in vitro expanded cultures of multiple subjects with type 1 diabetes. Representative tetramer staining for a GAD65_121–140_ and a GAD65_250–266_ clone is shown in Fig. 2A. These DQ8 tetramer-sorted clones were antigen-specific and the responses could be blocked by anti-DQ (SPVL3) antibodies (Fig. 2B). The GAD65-specific cells were assayed for cytokine production by intracellular cytokine staining (ICS) (Fig. S1A). All GAD65_250–266_-specific clones produced Th1-type cytokine IFN- γ (Fig. 2C). One GAD65_250–266_-specific clone also produced IL-4, but production of IFN- γ was still dominant in these cells (Fig. S1B). IL-17 was not detected in GAD65_250–266_ cells. The ICS data were in agreement with the cytokine ELISA results as IFN- γ was the main cytokine secreted from GAD65_250–266_ clones after peptide specific stimulation (Fig. 2D). Conversely, the cytokine profile for the four GAD65_121–140_-specific T clones was more diversified. Two GAD65_121–140_-specific clones isolated from different patients produced IL-17 (Fig. 2C). One of the IL-17-producing cells exhibited a Th1/Th17 double positive phenotype by co-expressing IFN- γ. Production of IL-17 was not a result of in vitro priming of naïve cells, as cells isolated from the memory population (CD45RA-CD4+) maintained the Th17-like phenotype (data not shown). The two IL-17-negative GAD65_121–140_-specific clones were positive for IFN- γ, IL-4, and IL-10. However, these two clones might have distinct functionality as suggested by the ratio of IFN- γ to IL-4 producing cells (Fig. S1B) and the cytokine ELISA results (Fig. 2D). Although multiple clones isolated from one donor might be the same T cell captured at different stages of differentiation, our T cell clone data from different patients suggest that DQ8-restricted GAD65-specific epitopes could induce proinflammatory responses in T1D subjects.

### Higher frequencies of GAD65_250–266_-specific T cells are observed in subjects with type 1 diabetes

**Figure 3:**
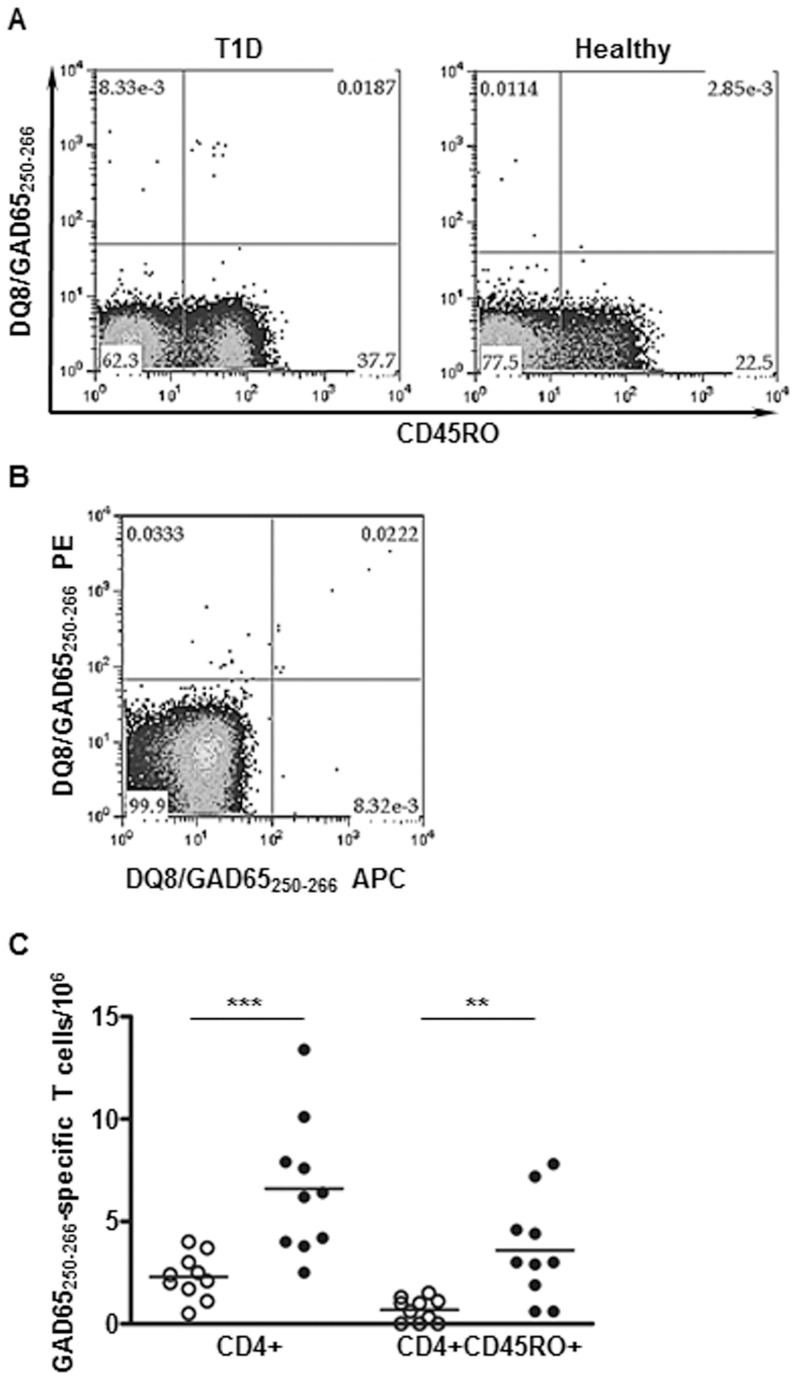
Direct ex vivo analysis of GAD65-specific T cells. Unmanipulated PBMCs were stained with DQ8/GAD65_250–266_ PE-tetramer. Antigen-specific CD4+ T cells were enriched, stained with antibodies against surface markers of interest, and analyzed on a Calibur multi-color flow cytometer. (**A**) Representative ex vivo analysis of the surface memory marker CD45RO for GAD65_250–266_. The frequency of GAD65_250–266_-specific CD45RO+CD4+ T cells was 4.4 per million CD4+ T cells for the T1D patient (left panel) and 0.6 per million for the healthy subject (right panel). (**B**) Ex vivo co-staining of GAD65_250–266_-specific cells with DQ8/GAD65_250–266_ PE- and DQ8/GAD65_250–266_ APC-tetramers. Cells were stained with PE-labeled DQ8/GAD65_250–266_ tetramers first. After enrichment, tetramer-positive cells were stained again with APC-labeled DQ8/GAD65_250–266_ tetramers at 37°C for 1 h. (**C**) Cumulative total CD4+ and CD45RO+CD4+ T cell frequencies for GAD65_250–266_ in controls (open circles, n = 10) and T1D patients (closed circles, n = 10). *** P<0.001, ** P<0.01, as evaluated by Mann-Whitney U-test.

In vitro responses can be affected by numbers and activity of monocytes and lymphocytes in the blood samples. To confirm the disease association of DQ8/GAD65-specific cells in an unmanipulated state, ex vivo tetramer staining was performed for DQ8-specific T cells in DQ8+ patients and DQ8+ controls (average age for patients: 28.8, and for controls: 34.6, *p* = 0.2809). Direct ex vivo analysis was able to detect GAD65_250–266_-specific CD4+ T cells in DQ8+ subjects, but not for GAD65_121–140_-specific cells (Fig. S2A and S2B). The unsuccessful detection might be due to low avidity or low frequencies of T cells specific for DQ8/GAD65_121–140_. A representative ex vivo staining for DQ8/GAD65_250–266_ from one T1D and one healthy subject is shown in Fig. 3A. The ex vivo staining for DQ8/GAD65_250–266_ is specific as these cells can be co-stained by DQ8/GAD65_250–266_ tetramers labeled with two different fluorochromes (PE or APC) (Fig. 3B).

Results of the ex vivo experiments are summarized in Table 3. The average frequencies of GAD65_250–266_-specific cells and GAD65_250–266_-specific CD45RO+ cells were found to be higher in the T1D group compared to the healthy control (Fig. 3C). There was no correlation between frequencies of GAD65_250–266_-specific CD45RO+ cells and disease duration (*r* ^2^ = 0.0218).

**Table 3:** Summary of ex vivo results for GAD65_250–266_.

T1D								
Age	Duration (Days)	Frequency per 10^6^ cells		% Memory	IAA	GADA	IA-2A	ZnT8A
		CD4+	CD4+CD45RO+					
14	1632	2.5	0.6	24.0	-	-	-	-
14	2439	3.8	0.6	15.8	+	-	-	-
17	1190	13.4	7.8	58.2	N/A	-	+	N/A
24	1379	6.2	2.9	46.8	+	+	+	+
25	292	7.9	3.0	38.0	-	+	+	+
25	1247	7.6	4.6	60.5	+	+	-	+
30	6564	4.2	1.9	45.2	-	-	+	+
41	1094	4.0	3.0	75.0	-	+	-	-
42	281	6.4	4.4	68.8	-	+	+	+
56	7507	10.1	7.2	71.0	+	+	-	-

Healthy							
Age	Frequency per 10^6^ cells		% Memory	IAA	GADA	IA-2A	ZnT8A
	CD4+	CD4+CD45RO+					
21	3.7	1.5	40.5	-	-	-	-
29	2.5	1.3	52.0	-	-	-	-
29	2.1	0.0	0.0	-	+	-	-
33	2.0	1.0	50.0	-	-	-	-
33	3.0	0.6	20.0	-	-	-	-
33	2.4	0.0	0.0	-	-	-	-
35	1.7	1.1	64.7	-	-	-	-
35	4.0	1.0	25.0	-	-	-	-
42	1.1	0.0	0.0	-	-	-	-
56	0.5	0.3	60.0	-	-	-	-

N/A: not available.

## Discussion

DQ8 possesses a predisposing non-aspartic acid residue at beta 57 and confers the highest risk to T1D. Substantial efforts have been made to identify DQ8-restricted responses which can closely monitor the progression of diabetes in human. However, detection of disease-related reactions from the rare islet-specific T cell populations is extremely challenging. In this study, we demonstrated DQ8 tetramers as a tool to study islet-specific T cell reactivity and identified two disease-associated peptides, GAD65_121–140_ and GAD65_250–266_ (Table 2, Fig. 2). In addition, T cells specific for GAD65_250–266_ possessed several attributes that render these cells a potential biomarker for T1D. GAD65_250–266_-specific cells exhibited robust responses upon peptide stimulation (Fig. 1), and could be detected directly in unmanipulated PBMCs (Fig. 3A). Most importantly, differences between patients and healthy subjects in frequencies of CD4+ and CD4+CD45RO+ cells specific for GAD65_250–266_ could readily be seen by ex vivo analysis (Fig. 3C). Monitoring of GAD65_250–266_-specific T cells can potentially provide new insights into the etiology and treatment of the disease, although further longitudinal studies to link specific responses to the beta cell function are essential to confirm its suitability as a biomarker.

Schneider et al. suggest that self-reactive T effector cells are less resistant to peripheral regulation in T1D patients [20]. The current GAD65_121–140_- and GAD65_250–266_-reactive T cell data support this model as responses to both peptides were more prevalent in T1D patients compared to controls (Fig. 1). Higher numbers of total antigen specific cells and CD4+CD45RO+ cells for GAD65_250–266_ (Fig. 3C) were also found in the periphery of subjects with T1D by ex vivo tetramer staining. Other studies show that DRB1*04:01 (DR0401)-restricted GAD65-reactive T cells of certain specificities are preferentially activated in T1D patients while other specificities are not [21]–[24]. The discrepancy in GAD65 responses to peripheral regulation might reflect that self-reactive T cells for different specificities are subjected to various degree of regulation. Further study in examining whether discrepancy in regulation of DR0401 and DQ8 restricted responses will be of interest. Previous studies show some islet-reactive T cells exhibit protective phenotype in healthy subjects [25], [26]. The presence of multiple effector properties for GAD65_121–140_ (Fig. 2C, Fig. 2D, and Fig. S1B) implies the potential of GAD65_121–140_–specific cells to modulate the immune balance between tolerance and autoimmunity. Future studies to compare the dynamics of T cell responses for each HLA haplotype during disease development will help to shed more insight into the immunopathology of T1D.

The presence of self-reactive T cells in healthy individuals highlights the importance of peripheral regulation as a checkpoint in the progression of T1D. The higher frequencies of GAD65_250–266_-specific total and memory CD4+ T cells in subjects with type 1 diabetes (Fig. 3C) suggest that quantity and phenotypic changes for self-reactive T cells might be an indicator for loss of tolerance. DQ8 tetramers can therefore be a useful tool for the identification of disease-related self-reactive T cells as reliable biomarkers. Monitoring of these cells could assist with early-detection of risk or progression to T1D. In conclusion, DQ8-restricted CD4+ T cells specific for GAD65_121–140_ and GAD65_250–266_ were detected in multiple T1D subjects. GAD65_121–140_- and GAD65_250–266_-reactive T cells were more prevalent in T1D patients. Further evaluation of T cell responses for beta cell autoantigens should facilitate our understanding of the role of autoreactive DQ8 T cells in type 1 diabetes and selection of autoreactive DQ8 T cells as T1D biomarkers.

## Materials and Methods

### Subjects

The studies were approved by the Institutional Review Board of Benaroya Research Institute (BRI, Seattle, WA). All DQ8+ subjects were volunteers of Caucasian descent and were recruited with written consent from participating individuals or their guardians. HLA typing was conducted by BRI sequencing and genotyping core facilities. Samples from DQ8-positive T1D (n = 33, age from 9 to 56, days of disease duration from 281 to 7507) or DQ8-positive individuals without T1D or other autoimmune disease (healthy controls, n = 23, age from 18 to 56) were obtained under the auspices of the BRI JDRF Center for Translational Research at Benaroya Research Institute and Seattle Children's.

### DQ8 protein and tetramers

Recombinant DQ8 protein was produced as previously described [27]. Briefly, soluble DQ8 was purified from insect cell culture supernatants by affinity chromatography and dialyzed against citric/phosphate storage buffer, pH 5.4. For the preparation of HLA class II tetramers, DQ8 protein was in vivo biotinylated in Drosophila S2 cells [28] prior to harvest and dialyzed into citric/phosphate buffer. The biotinylated monomer was loaded with 0.2 mg/ml of peptide by incubating at 37°C for 72 h in the presence of 0.2 mg/ml n-Dodecyl-β-maltoside and 1 mM Pefabloc SC (Sigma–Aldrich, St. Louis, MO). Peptide loaded monomers were subsequently conjugated into tetramers using R-PE streptavidin (Biosource International, Camarillo, CA) at a molar ratio of 8∶1.

### Peptides

Peptides derived from GAD65 (Table 1) were synthesized by Mimotopes (Clayton, Australia). The biotinylated reference peptide GAD65_250–266_ (AMMIARFKMFPEVKEKG) was synthesized by Genscript with one 6-aminohexanoic acid spacer added between the N-terminal biotin label and the remainder of the peptide sequence.

### Peptide binding competition

GAD65_250–266_ is a good binder for DQ8 [29] and was used as the index peptide in the competition study. Various concentrations of each test peptide were incubated in competition with 0.04 µM biotinylated GAD65_250–266_ peptide in wells coated with DQ8 protein. After washing, the remaining biotin-GAD65_250–266_ peptide was labeled using europium-conjugated streptavidin (Perkin Elmer) and quantified using a Victor2 D time resolved fluorometer (Perkin Elmer). Peptide binding curves were simulated by non-linear regression with Prism software (Version 5, GraphPad Software Inc.) using a sigmoidal dose–response curve. IC_50_ binding values were calculated from the resulting curves as the peptide concentration needed for 50% inhibition of reference peptide binding.

### Self-reactive T cell identification

PBMCs were prepared from the blood of T1D or healthy DQ8 subjects by Ficoll underlay. CD4+ T cells were isolated using the Miltenyi CD4+ T cell isolation kit, plated in 48-well plates with APCs, and stimulated with 20 µg/ml peptides from the selected peptide set. The cells were cultured for two weeks in the presence of T cell medium (RPMI-1640 (Gibso) +10% pooled human serum +1% penicillin-streptomycin) and IL-2 (Hemagen Diagnostics, Inc.) at 37°C, stained with PE-tetramers and antibodies of interest, and analyzed by flow cytometry. 20000 total events were collected for each staining. Lymphoid cells were gated based on forward and side scatter profile. The negative threshold for gating for Fig. 1 was set based on the staining of samples with empty tetramers. Our criterion for positivity was distinct staining that labeled a compact tetramer positive CD4+ population, and was more than three-fold above background (cells from an unstimulated well) in the same experiment.

### *Ex vivo* analysis of self-reactive T cells

Ex vivo tetramer staining was performed as previously described [30]. Briefly, 30 million PBMCs in culture medium at a concentration of 150 million/mL were incubated with 50 nM dasatinib (LC Laboratories, dissolved in DMSO) for 10 min at 37°C. The cells were next stained with 20 µg/mL phycoerythrin (PE)–labeled tetramers at room temperature for 120 minutes, followed by antibody staining for 20 minutes at 4°C with CD4 APC (clone RPA-T4, eBioscience), CD45RO FITC (clone UCHC1, eBioscience), and a combination of CD14 PerCP (clone MφP9, BD PharMingen) and CD19 PerCP (clone SJ25C1, BD PharMingen) to exclude B cells and monocytes from the analysis. Cells were washed twice with FACS running buffer (0.5% BSA and 2mM EDTA in PBS) and incubated with anti-PE magnetic beads (Miltenyi Biotec, Bergisch Gladbach, Germany) at 4°C for another 20 minutes. The cells were washed again and enriched with a Miltenyi MS magnetic column. Samples were labeled with ViaProbe (BD Biosciences) and analyzed on a BD FACS Calibur flow cytometer. Frequencies were calculated by dividing the number of tetramer positive cells in the bound fraction by the number of total CD4+ T cells in the sample.

### Statistical analysis

Statistical analysis was performed with Prism software (Version 5.03, GraphPad Software Inc.). The Mann-Whitney U-test was used to preform two group comparisons of T cell frequency. Fisher's exact test was used to compare the prevalence of T cell responses in T1D and healthy subjects.

### T cell clone isolation

T cell clones were generated by staining cultured T cells with tetramers, sorting gated tetramer-positive CD4+ cells using a FACS Aria (at single-cell purity, applying a singlet gate to the lymphocyte population) and expanding in a 96-well plate in the presence of 1.0×10^5^ irradiated PBMCs and 2 µg/ml phytohemagglutinin (Remel Inc. Lenexa, KS). After expansion of each T cell clone to a single 48 well, clones were stained again with tetramer and also stimulated in parallel with the corresponding peptides (10 µg/mL), adding HLA-DQ-matched irradiated PBMCs as antigen presenting cell and measuring thymidine uptake to verify epitope specificity.

### Intracellular cytokine staining

Autoreactive-T cell clones isolated from T1D patients were resuspended in 200 µl of T cell medium (RPMI-1640 (Gibco) +10% pooled human serum +1% penicillin-streptomycin), and stimulated with 50 ng/mL phorbol 12-myristate 13-acetate and 1 µg/mL ionomycin in the presence of 10 µg/mL Brefeldin A for 4 hours at 37°C. After incubation cells were stained with surface antibodies including CD3 PE-Cy5 (clone HIT3a, BioLegend) and CD4 v500 (clone RPA-T4, BD Biosciences) as well as Fixable Viability Stain 450 (BD Horizon). Cells were then fixed and permeabilized as per the manufacturer's instructions (eBioscience). Cells were next stained with antibodies against IFN-γ AF700 (clone 4S.B3, BioLegend), IL-10 PE Cy7 (clone MQ1-17H12, BioLegend), IL-17A APC Cy7 (clone BL168, BioLegend), and IL-4 FITC (clone 8D4-8, eBioscience) for 20 minutes at 4°C. Cells were then washed in PBS and immediately analyzed by flow cytometry on a BD LSRII multi-color flow cytometer. Clones were considered cytokine positive if more than 10% of the cells produced that particular cytokine.

## Supporting Information

Figure S1 **Intracellular cytokine staining for DQ8-specific T cell clones.** T cell clones specific for GAD65_121–140_ and GAD65_250–266_ were stimulated with 50 ng/mL phorbol 12-myristate 13-acetate and 1 mg/mL ionomycin in the presence of 10 mg/mL Brefeldin A in 1 mL of T cell medium for 4 hours at 37°C. Cells were fixed, permeabilized, stained with antibodies for IFN- γ, IL-10, IL-4, and IL-17, and analyzed on a LSRII multicolor flow cytometer. (**A**) Representative intracellular cytokine staining analysis for the GAD65_250–266_ T cell clone T1D01-C6. Staining results (open histograms) were compared to cells incubated with nonspecific isotype matched IgG control antibodies (gray histograms). Numbers indicate the percentage of cytokine-producing cells. (**B**) The ratio of IFN- γ producing cells to IL-4 producing cells in IFN- γ ^+^ IL-4^+^ clones for GAD65_121–140_ and GAD65_250–266_. Each dot represents one IFN- γ ^+^IL-4^+^ clone from Fig 2(C) (T1D05-C2 and T1D05-C3 for GAD65_121–140_, and T1D01-C6 for GAD65_250–266_, respectively).
(TIF)

Figure S2 **Direct ex vivo flow cytometric analysis of DQ8/GAD65-specific T cells.** (**A**) Gating strategy for Tetramer+ cells. Lymphoid cells were selected based on forward and side scatter profile. A dump channel was used to exclude monocytes (CD14+), B cells (CD19+), and dead cells (ViaProbe) from lymphocytes. Viable Tetramer+CD4+CD45RO+ T cells were gated based on the staining of unenriched cells, and the gating was applied to enriched populations. Numbers indicate the percentage of cells in the gated regions or each quadrant. (**B**) Representative ex vivo analysis of the surface memory marker CD45RO for GAD65_121–140_-specific cells in a T1D subject. The frequency of these cells is below the threshold of detection.
(TIF)
