# Supplementary figures and images for: Assessment of CD4+ T Cell Responses to Glutamic Acid Decarboxylase 65 Using DQ8 Tetramers Reveals a Pathogenic Role of GAD65 121–140 and GAD65 250–266 in T1D Development
Source: PLoS One. 2014 Nov 18;9(11):e112882. doi: 10.1371/journal.pone.0112882 (PMC4236121; doi:10.1371/journal.pone.0112882)

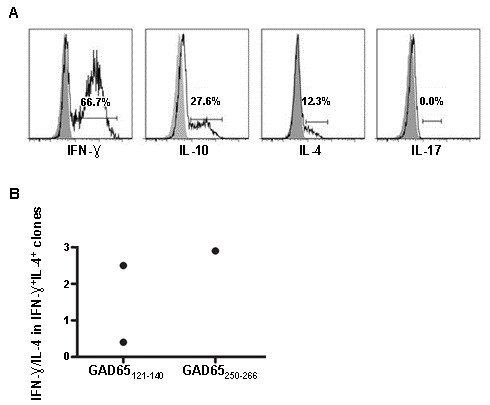

Supplement: Figure S1 — Intracellular cytokine staining for DQ8-specific T cell clones. T cell clones specific for GAD65121–140 and GAD65250–266 were stimulated with 50 ng/mL phorbol 12-myristate 13-acetate and 1 mg/mL ionomycin in the presence of 10 mg/mL Brefeldin A in 1 mL of T cell medium for 4 hours at 37°C. Cells were fixed, permeabilized, stained with antibodies for IFN- γ, IL-10, IL-4, and IL-17, and analyzed on a LSRII multicolor flow cytometer. (A) Representative intracellular cytokine staining analysis for the GAD65250–266 T cell clone T1D01-C6. Staining results (open histograms) were compared to cells incubated with nonspecific isotype matched IgG control antibodies (gray histograms). Numbers indicate the percentage of cytokine-producing cells. (B) The ratio of IFN- γ producing cells to IL-4 producing cells in IFN- γ + IL-4+ clones for GAD65121–140 and GAD65250–266. Each dot represents one IFN- γ +IL-4+ clone from Fig 2(C) (T1D05-C2 and T1D05-C3 for GAD65121–140, and T1D01-C6 for GAD65250–266, respectively). (TIF) [file pone.0112882.s001.tif]

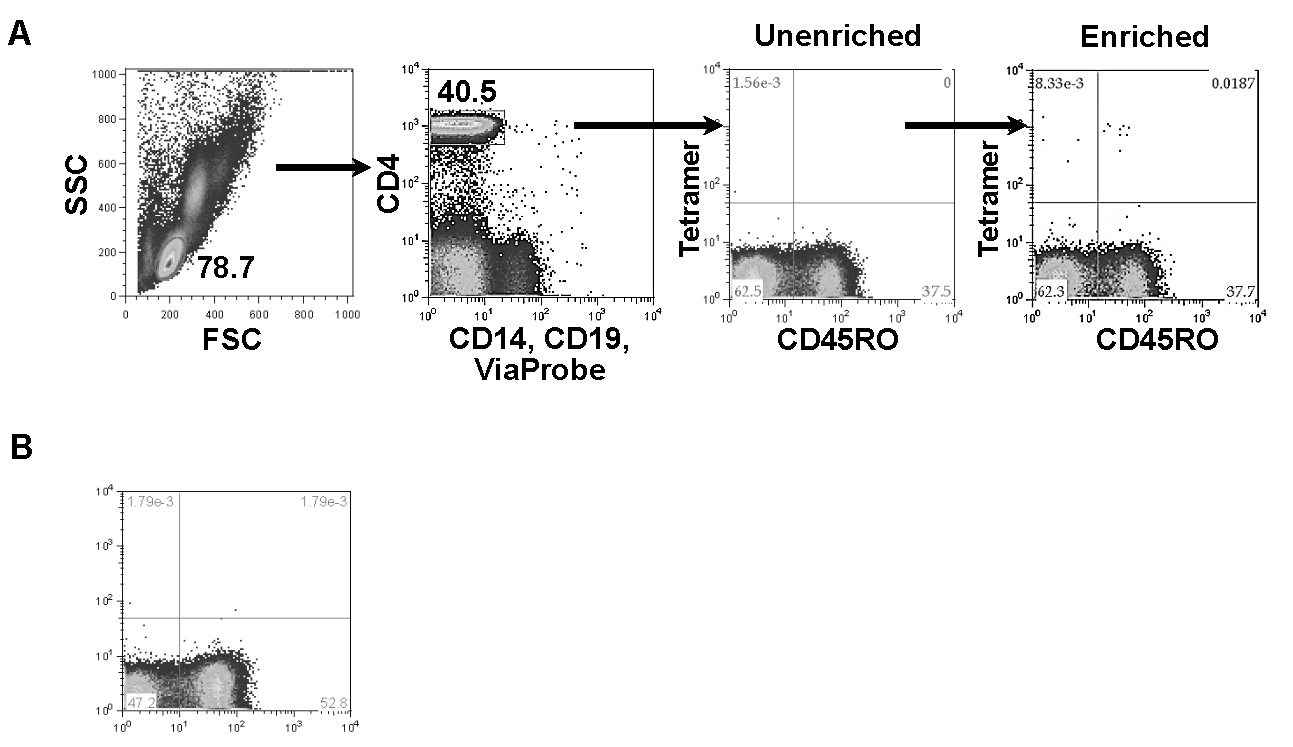

Supplement: Figure S2 — Direct ex vivo flow cytometric analysis of DQ8/GAD65-specific T cells. (A) Gating strategy for Tetramer+ cells. Lymphoid cells were selected based on forward and side scatter profile. A dump channel was used to exclude monocytes (CD14+), B cells (CD19+), and dead cells (ViaProbe) from lymphocytes. Viable Tetramer+CD4+CD45RO+ T cells were gated based on the staining of unenriched cells, and the gating was applied to enriched populations. Numbers indicate the percentage of cells in the gated regions or each quadrant. (B) Representative ex vivo analysis of the surface memory marker CD45RO for GAD65121–140-specific cells in a T1D subject. The frequency of these cells is below the threshold of detection. (TIF) [file pone.0112882.s002.tif]
